# Scrub Typhus Meningoencephalitis: An Overlooked Entity

**DOI:** 10.7759/cureus.28989

**Published:** 2022-09-09

**Authors:** Ashutosh Upadhyaya, Mohammad R Alam, Ali Akbar Raeen, Shriya Upadhyaya, Monika Pathania, Susmita Upadhyaya, Kumarasamy Sivanu

**Affiliations:** 1 Medicine, Evergreen Hospital Pvt. Ltd, Parasi, NPL; 2 Internal Medicine, Argakhachi District Hospital, Argakhachhi, NPL; 3 Internal Medicine, Institute of Medicine (IOM) Tribhuvan University Teaching Hospital (TUTH), Kathmandu, NPL; 4 Internal Medicine, All India Institute of Medical Sciences, Rishikesh, IND; 5 Internal Medicine, Milton Keynes University Hospital, University of Buckingham, Buckingham, GBR

**Keywords:** orientia tsutsugamushi, acute encephalitis syndrome, tropical fever, meningoencephalitis, scrub typhus

## Abstract

Scrub typhus is common in rural parts of Nepal, but its diagnosis remains difficult due to a lack of clinical suspicion and poor diagnostic resources. The absence of common clinical features further complicates this problem. Acute kidney injury (AKI), myocarditis, rhabdomyolysis, hepatitis, acute respiratory distress syndrome, and meningoencephalitis are complications of the disease associated with high mortality. Overlap findings can be noted in scrub typhus meningoencephalitis and other tropical infections. This makes diagnosing the disease more challenging, especially in areas where the burden of infectious diseases is high. We report three cases of scrub typhus meningoencephalitis. All three patients were treated successfully with doxycycline. Because patients with scrub typhus have an excellent response to treatment, delay in treatment and rate of complications can be prevented with high clinical suspicion of the condition.

## Introduction

Scrub typhus is caused by *Orientia tsutsugamushi*, a trombiculid mite-borne bacterium replicating in endothelial cells and phagocytes. Eschar is the characteristic lesion that starts as a vesicular lesion at the site of mite feeding. Later, an ulcer forms with a black necrotic center, an erythematous border, and regional lymphadenopathy. Other features are fever and maculopapular rash starting from the trunk and spreading to the limbs. It has a predilection for highly vascularized organs such as the brain, heart, lungs, and liver causing severe complications like myocarditis, pneumonia, meningoencephalitis, acute renal failure, gastrointestinal bleeding, and acute respiratory distress syndrome. Up to one-fifth of patients have significant nervous system involvement [1]. In endemic areas of the Indian subcontinent, scrub typhus is increasingly being reported as the cause of meningoencephalitis [2-3]. A lack of research and investigation into scrub typhus has left Nepal vulnerable to the disease. Several suspected instances were recorded between 2004 and 2014, but none were verified. An epidemic was suspected following a catastrophic earthquake in 2015, which prompted the reporting of 101 confirmed cases across 16 districts [4]. An increasing trend in the following year led to the declaration of an epidemic.

Meningitis and encephalitis are the most common neurological manifestations, while opsoclonus, myoclonus, parkinsonism, and Guillain-Barre syndrome (GBS) are less common presentations reported in the literature [5]. Neurologic dysfunction, including neck stiffness, neurologic weakness, seizures, delirium, and coma, have been described in patients with severe central nervous system involvement. Meningismus or meningitis has been found in nearly 5.7%-13.5% of patients [6]. Pathognomic eschar, a sign of chigger bite, may not always be found, and its absence is associated with worse outcomes [7].

Variable presentation of the disease is a known cause of delay in diagnosis. In our patients, initially, tuberculous and bacterial meningitis were amongst the main differentials for each of the cases. In addition, the diagnostic tests specific to scrub typhus are either unavailable in most parts of the developing world or inadequate. These factors frequently delay the identification of scrub typhus meningitis and encephalitis, resulting in a greater fatality rate [1]. We present three cases of scrub typhus meningoencephalitis.

## Case presentation

All three cases have been described briefly, followed by a table containing relevant details. 

Case 1

A 52-year-old female farmer, resident of Gulmi district (hilly region of Nepal), with no prior comorbidities, complained of moderate-grade intermittent fever with myalgia for five days and confusion for one day. She had hypoactive delirium and could not recognize any of her relatives. There was no history of headache, rash, vomiting, trauma, or seizure. There was no history of cough, hemoptysis, jaundice, abdominal pain, constipation or diarrhea, oliguria, or dysuria. There was no recent travel history, and vaccination history was unavailable. Clinical examination was relevant for pallor, confusion, meningismus, right-sided peripheral facial nerve palsy, and dysarthria. Fundoscopy was negative for papilledema. Non-tender hepatosplenomegaly was noted, but there was no evidence of insect bite marks or eschars (Table 1). After sending the blood samples for a baseline evaluation, the patient was initially treated with empirical antibiotic therapy for bacterial meningitis. Non-contrast computed tomography (NCCT) of the brain was obtained, which showed a normal study. Her laboratory results revealed leukocytosis with neutrophilia, thrombocytopenia, and hepatic and renal dysfunction. The blood culture did not grow any organism. Lumbar puncture was performed, and cerebrospinal fluid (CSF) analysis showed lymphocytic pleocytosis and high CSF adenosine deaminase. Serology for locally prevalent tropical infections was ordered and was positive for scrub typhus immunoglobulin M (IgM)/indirect immunofluorescence assay (IFA). Evaluation for other tropical infections was negative (Table 2). The empirical antibiotic therapy was switched to oral doxycycline, and the patient showed significant clinical improvement and recovered without any neurological sequelae (Table 1).

**Table 1:** Case presentation.

Particulars	Case 1	Case 2	Case 3
Age and gender	52 years, female	19 years, female	47 years, female
Presenting complaints	Fever, myalgia, and altered sensorium	Fever, vomiting, lower extremity edema, altered sensorium	Fever, shortness of breath, altered sensorium
Eschar	Absent	Absent	Absent
Travel history	Not any	Not any	Not any
Vaccination history	Unavailable	Fully vaccinated per the national immunization schedule	Unavailable
Resident of (height above sea level)	Gulmi district, Nepal (610-3050 m)	Arghakhachi district, Nepal (302-2515 m)	Gulmi district, Nepal (610-3050 m)
Duration of symptoms at presentation to the hospital	5 days	10 days	12 days
Neurologic findings	Neck rigidity - present	Neck rigidity - present	Neck rigidity - absent
	Bilateral plantar reflex-flexor response	Bilateral Plantar reflex - extensor response	Bilateral plantar reflex - extensor response
	Right-sided facial (upper and lower) weakness and incomprehensible speech	No facial weakness and normal speech	No facial weakness and normal speech
Fundoscopic findings	No papilledema	No papilledema	No papilledema
Other findings	Pallor	Pallor	Tachycardia
	Hepatosplenomegaly	Icterus	Hypotension
		Hepatosplenomegaly	
	Treatment and response	Oral Doxycycline for 5 days	Oral doxycycline for 5 days	IV Doxycycline for 5 days
Resolution of symptoms in 3 days		Resolution of symptoms in 5 days	Resolution of symptoms in 5 days

**Table 2:** Laboratory findings. IgM, immunoglobulin M, IFA, indirect immunofluorescence assay; ICT, immunochromatographic test, Gen., generation; ELISA, enzyme-linked immunosorbent assay; CSF, cerebrospinal fluid; NCCT, non-contrast computed tomography; AST, aspartate transaminase; ALT, alanine transaminase

Parameters	Case 1	Case 2	Case 3	Reference values
Complete blood count				
Total leukocyte count	14900/mm^3^	15,300/mm^3^	19,400/mm^3^	4000-10,000/mm^3^
Neutrophil	74%	68%	78%	40%-70%
Platelet	80,000/mm^3^	65,000/mm^3^	150,000/mm^3^	150,000-400,000/mm^3^
Hemoglobin	9.8 g/dL	12.1g/dl	11.4 g/dL	13-17 g/dL
Liver function test				
Total bilirubin	1.34 mg/dL	1.9mg/dl	1.6 mg/dL	0.1-1.2 mg/dL
Aspartate transaminase	101 IU/L	170 IU/L	210 IU/L	0-50 IU/L
Alanine transaminase	84 IU/L	143 IU/L	186 IU/L	0-50 IU/L
Alkaline phosphatase	177 IU/L	220 IU/L	243 IU/L	45-120 IU/L
Renal function test				
Sr. urea	19 mg/dL	148mg/dl	56 mg/dL	17-43 mg/dL
Sr. creatinine	1.8 mg/dL	4mg/dl	2.3 mg/dL	0.7-1.2 mg/dL
Serology				
Scrub typhus IgM (IFA/ICT)	1:200 (IFA)	1:200 (IFA)	Positive (ICT)	
Malarial parasite (ICT)	Negative	Negative	Negative	
Dengue IgM and IgG (ELISA)	Negative	Negative	Negative	
HIV (4^th^ Gen. ELISA)	Negative	Negative	Negative	
Leptospirosis IgM (ELISA)	Negative	Negative	Negative	
CSF findings				
Appearance	Clear	Clear	(Not done)	
Opening pressure	10 cm of H20	10 cm of H20		
Total leukocyte count	120 cells/mm^3^	115 cells/mm^3^		
Polymorphs	20%	27%		
Lymphocyte	83%	78%		
Protein	89 mg/dL	102 mg/dL		
Sugar	67 mg/dL	73 mg/dL		
Adenosine deaminase	12.4 U/L	10.8 U/L		
NCCT brain	No significant findings	No significant findings	(Not done)	

Case 2

A 19-year-old female student from Arghakhanchi district (hilly region of Nepal), with no prior comorbidities, presented with a complaint of moderate-grade intermittent fever for 10 days prior to presenting to our hospital. There was no associated headache, rash, cough, abdominal pain, constipation or diarrhea, oliguria, or dysuria. She also had edema in her lower limbs for five days that was gradually progressive, not associated with dyspnea, orthopnea, frothy urine, or oliguria. She had two to three episodes of vomiting for two days that were non-projectile, non-bilious, non-blood stained, and contained food particles. She developed confusion for a day in the later course of the disease. She was agitated, had hyperactive delirium, and could not recognize her relatives. There was no recent travel history, and the patient was fully vaccinated per the national immunization schedule. On examination, she was pale, afebrile, and hemodynamically stable. She was not oriented to time, place, and person. Neck rigidity was present, but there was no papilledema. Non-tender hepatosplenomegaly was also noted. There was no evidence of any insect bite marks or eschar (Table 1). After sending the relevant blood samples, the patient was initially treated with empirical antibiotic therapy for bacterial meningitis. NCCT of the brain was obtained, and it was a normal study. Her laboratory parameters revealed leukocytosis and thrombocytopenia with hepatic and renal dysfunction. The blood culture did not grow any organism. Lumbar puncture was performed, and CSF analysis showed lymphocytic pleocytosis and high CSF adenosine deaminase. Serology for locally prevalent tropical infections was ordered and was positive for scrub typhus IgM/IFA. Evaluation for other tropical infections was negative (Table 2). The empirical antibiotic therapy was switched to oral doxycycline, and the patient showed significant clinical improvement (Table 1).

Case 3

A 47-year-old female farmer, resident of Gulmi district (hilly region of Nepal), with no prior comorbidities, presented with complaints of low-grade intermittent fever and myalgia for 12 days. It was not associated with headache, vomiting, rash, cough, abdominal pain, constipation or diarrhea, oliguria, or dysuria. She also had acute-onset dyspnea for two days with a Modified Medical Research Council (MMRC) grade of two, with no associated orthopnea, cough, or oliguria. She also developed an altered mental state for two days in the later course of the disease. She had hyperactive delirium, was self-muttering, and could not recognize any of her relatives. There was no recent travel history, and vaccination history was unavailable. On examination, she was pale, dehydrated, febrile with a 102°F axillary temperature, hypotensive, and had tachycardia. She was not oriented to time, place, and person. There was neck rigidity. There was no evidence of insect bite marks or eschars on her body (Table 1). The chest X-ray, electrocardiogram, and D-dimer were normal. Her laboratory parameters evidenced leukocytosis with hepatic and renal dysfunction. The blood culture did not grow any organism. Because of financial constraints, CSF analysis and CT brain could not be done for the patient. Serology for locally prevalent tropical infections was ordered and was positive for scrub typhus IgM/ICT. Evaluation for other tropical infections was negative (Table 2). The empirical antibiotic therapy was switched to IV doxycycline, to which the patient responded rapidly. She was discharged in a hemodynamically stable condition with oral doxycycline (Table 1). 

## Discussion

Scrub typhus has been increasingly reported in the southern plains of Nepal in the last few years. People living in earthen floor homes are particularly at high risk. Occupational or agricultural settings associated with the disease include exposure to rice fields, bushes, stacks of wood, rodents, and farm animals [8]. Common symptoms of the disease overlap with other tropical illnesses like malaria, enteric fever, dengue, brucellosis, and leptospirosis. Elevated hepatic enzymes, deranged renal function, and thrombocytopenia are also seen in most patients with occasional leukopenia or leukocytosis [9]. Systemic vasculitis and perivasculitis can lead to severe complications such as acute respiratory distress syndrome, meningoencephalitis, pneumonitis, acute renal failure, and myocarditis, which infrequently occur but can be life-threatening. Pneumonia, delirium, and myocarditis are associated with increased mortality [3]. Eschar was previously considered pathognomic, but it is seen in 7%-68% of cases. The absence of eschar is a risk factor for mortality, which can be attributed to delays in treatment, causing the onset of complications [7]. Even after extensive examination, no eschar was found in all three of our cases.

A culture for scrub typhus takes four weeks to yield a positive result. Nested polymerase chain reaction (PCR) from the eschar samples is an excellent alternative for diagnosis. It can help in the early diagnosis of the disease within the first three days of fever onset, even before the appearance of antibodies [10]. The Centre for Disease Control (CDC) recommends documenting a four-fold rise in antibody titer between acute and convalescent samples to diagnose scrub typhus. Acute specimens are taken during the first week of illness, and convalescent samples are taken two to four weeks later. IFA is a reference standard, but other methods like immunochromatographic tests, enzyme-linked immunosorbent assay (ELISA), and indirect immunoperoxidase assays can also be used in places with limited resources [11].

The incubation period of the pathogen is 7-10 days, after which patients present with symptoms like fever, headache, arthralgia, myalgia, and cough. Complications such as acute kidney injury (AKI), encephalitis, and acute respiratory distress syndrome (ARDS) usually develop later. The mortality rate is also higher when patients present with a relatively early onset of these complications [12]. Kidney involvement has been reported in the background of meningoencephalitis caused by scrub typhus. A study by Kar et al. emphasizes the need to consider the possibility of scrub typhus in all patients with acute encephalitis syndrome with renal involvement [13]. Consistent with the findings of this study, all three of our patients had renal dysfunction. Vasculitis of renal vessels, third space fluid loss, and multi-organ dysfunction are all potential explanations for the AKI seen in scrub typhus infection. Liver dysfunction is known to occur in scrub typhus infection. A study by Hu et al. revealed a median aspartate aminotransferase (AST) of 148 U/L compared to median alanine aminotransferase (ALT) of 120 U/L [14]. All three of our patients had AST levels higher than ALT.

Acute encephalitis syndrome is characterized by a rapid onset of febrile illness associated with seizures, altered sensorium, and focal neurological deficits such as aphasia, hemiparesis, involuntary movements, ataxia, or cranial nerve palsies [15]. The mean duration of fever before a presentation is 7-8 days for scrub typhus meningoencephalitis, which is usually longer than what is seen in viral or bacterial encephalitis [5]. In our case, patients presented 5, 10, and 12 days after the onset of fever. Meanwhile, tuberculous meningitis is known to have an even longer mean duration of symptoms before the presentation. Headache is a common feature of scrub typhus infection generally, and severe holo-cranial headaches characterize those complicated by meningoencephalitis. Altered sensorium, seizures, and signs of meningeal irritation may be seen in up to 56%, 46%, and 49% of cases of meningoencephalitis, respectively [2]. Unilateral or bilateral sixth nerve palsy is a common finding in scrub meningoencephalitis, but bilateral facial nerve involvement has also been reported. Kim et al. reported a case of a patient presenting with both cranial nerve six and seven palsies [16]. One of our patients presented with a seventh cranial nerve deficit, while the others had normal cranial nerve exam findings. Cranial nerve involvement is also common in tuberculous meningitis and does not help in the clinical differentiation of the two diseases.

Conventional neuroimaging may show non-specific findings in scrub typhus meningoencephalitis. A study based on diffuse tensor imaging by Phukan et al. revealed alteration of subcortical white-matter integrity in scrub typhus meningoencephalitis representing axonal degeneration and myelin breakdown [17]. CSF analysis in scrub typhus meningoencephalitis is distinct from bacterial meningitis, as lymphocytic pleocytosis is not seen in the latter. But the findings remain mainly similar to those seen in tuberculous meningitis. CSF adenosine deaminase (ADA) of more than 10 U/L has a high sensitivity for tuberculous meningitis and may be used to differentiate the two diseases. Still, some studies have found it in the same range in scrub typhus meningoencephalitis [2]. In two of our patients in whom CSF analysis was done, CSF ADA was higher than the value suggested to be highly sensitive for tuberculous meningitis. No known history of tuberculosis infection, no contact, normal CT brain, relatively short disease onset, and positive scrub typhus serology made tuberculous meningitis less likely. When suspicion for tuberculous meningitis is high, detection of mycobacteria in CSF via smear microscopy or the nuclei acid amplification test (NAAT), or a combination of basal cistern enlargement on imaging and peripheral blood or CSF interferon-gamma release assay (IGRA), can aid in diagnosis [18].

The prognosis of neurological dysfunction is usually favorable. Only a few cases of prominent sequelae have been reported. All three of our patients recovered with no neurological sequelae. The mortality rate was 9% in a recently published large cohort study of 623 patients hospitalized with scrub typhus infection of varying severity, from mild to critically ill [3]. Meanwhile, a study from India reports a case fatality rate of 49% in encephalitis caused by scrub typhus [19]. Doxycycline for a minimum duration of five days or three afebrile days remains the standard of care. Despite the early use of doxycycline in scrub typhus meningoencephalitis, mortality has been reported owing to its poor penetration of the blood-brain barrier, poor gastrointestinal absorption, antibiotic resistance, and immune-mediated damage [20]. Injectable doxycycline or azithromycin can be a good alternative in such a situation, but their availability is yet another challenge, especially in resource-limited endemic areas.

## Conclusions

Based on our cases and literature review, we suggest that scrub typhus should always be considered a differential diagnosis in meningoencephalitis, especially when the onset of symptoms is subacute and liver and kidney function derangement is evident. Further study is needed to find the clinicopathological markers that could help differentiate scrub typhus meningoencephalitis from other types of meningitis.
